# A Comparison of the Immunometabolic Effect of Antibiotics and Plant Extracts in a Chicken Macrophage-like Cell Line during a *Salmonella* Enteritidis Challenge

**DOI:** 10.3390/antibiotics12020357

**Published:** 2023-02-08

**Authors:** Giulia Giovagnoni, Famatta Perry, Benedetta Tugnoli, Andrea Piva, Ester Grilli, Ryan J. Arsenault

**Affiliations:** 1 DIMEVET, Dipartimento di Scienze Mediche Veterinarie, Università di Bologna, Via Tolara di Sopra 50, Ozzano dell’Emilia, 40064 Bologna, Italy; 2 Department of Animal and Food Sciences, University of Delaware, 531 South College Ave., Newark, DE 19716, USA; 3 Vetagro S.p.A., Via Porro 2, 42124 Reggio Emilia, Italy; 4 Vetagro Inc., 17 East Monroe Street, Suite #179, Chicago, IL 60603, USA

**Keywords:** *Salmonella* Enteritidis, HD11, foodborne diseases, innate immunity, cellular metabolism, plant extracts, antibiotic alternatives

## Abstract

Immunometabolic modulation of macrophages can play an important role in the innate immune response of chickens triggered with a multiplicity of insults. In this study, the immunometabolic role of two antibiotics (oxytetracycline and gentamicin) and four plant extracts (thyme essential oil, grape seed extract, garlic oil, and capsicum oleoresin) were investigated on a chicken macrophage-like cell line (HD11) during a *Salmonella* Enteritidis infection. To study the effect of these substances, kinome peptide array analysis, Seahorse metabolic assay, and gene expression techniques were employed. Oxytetracycline, to which the bacterial strain was resistant, thyme essential oil, and capsicum oleoresin did not show any noteworthy immunometabolic effect. Garlic oil affected glycolysis, but this change was not detected by the kinome analysis. Gentamicin and grape seed extract showed the best immunometabolic profile among treatments, being able to both help the host with the activation of immune response pathways and with maintaining a less inflammatory status from a metabolic point of view.

## 1. Introduction

*Salmonella enterica* Serovar Enteritidis represents the most frequently reported serotype causing human salmonellosis in the United States [1]. According to the National Veterinary Services Laboratories (NVSL) of the USDA, chicken clinical and non-clinical samples represent, respectively, 50% and 82% of non-human isolates of *Salmonella* spp. collected from 1968 to 2011 [2]. Salmonellosis is a common foodborne disease that causes acute gastroenteritis in humans, and is frequently transmitted by raw poultry products [3]. Since the large majority of chickens infected with *Salmonella* Enteritidis are asymptomatic and, as a consequence, there is no clinical evidence of the infection, cross-contamination of products at the slaughterhouse is difficult to prevent [4]. Furthermore, the contamination of edible parts of chicken products (i.e., the liver) is not unusual if the bacterium invades enterocytes, translocates from the intestine, and spreads systemically [5]. To do so, the bacterium enters in macrophages, using the set of genes encoded in the Salmonella pathogenicity island 1 (SPI-1) [6], and takes advantage of these cells in order to survive, evade the immune system, and spread systemically in the bloodstream to the liver and the spleen [7,8]. Thus, macrophages play an important role in *Salmonella* pathogenesis. The role of these cells in innate immunity is to maintain tissue homeostasis in both physiological and pathological conditions [9]. In fact, under different stimuli, these cells have the ability to differentiate into pro-inflammatory M1 macrophages or anti-inflammatory M2 macrophages, producing different cytokines and chemokines, thus having opposing functions in the regulation of the immune response [9,10]. This is also due to metabolic reprogramming, switching to pathways that lead the differential phenotype of cells [10,11]. For example, the stimulation of Toll-like receptor (TLR) 4, induces the expression of hypoxia-inducible factor 1α (HIF1α) and promotes glycolysis in M1 macrophages, in order to promptly produce ATP and biosynthesize compounds for fighting bacterial infections [11,12]. On the other hand, M2 macrophages prefer oxidative phosphorylation in order to store energy and support their metabolism [11,12]. The role of macrophages is critical in the interaction of the bacterium and host, being the first line of defense of the host and, on the other hand, the Trojan horse of *Salmonella* for spreading within the organism. Chicken macrophage-like cells (HD11) represent a well-established in vitro model in order to study the interactions between macrophages and *Salmonella*. *S. enteritidis* can survive up to 8 h when in contact with HD11 cells and invade them [13,14]. Cells, in response to the bacterial infection, produce nitric oxide (NO) and induce the expression of *IL-1β*, *IL-6*, *IL-8*, and *IL-10* genes after 4 h [13]. This can be confirmed since, during the early stage of *S.* Enteritidis infection, cells increase the rate of glycolysis, suggesting a pro-inflammatory response [14].

The immunomodulatory properties of various classes of antibiotics have lately been under investigation. In fact, these drugs are not only able to inhibit or kill bacteria, but they also act on the host’s immune system [15], ideally explaining part of their role as growth promoters. For example, the class of tetracyclines and aminoglycosides exhibited immunomodulatory effects in various fields of research [16,17,18,19,20]. The growing demand for alternatives to antibiotics has led to the study of different plant extracts and their properties. The great variety of sources of plant extracts reflects the wide range of biological activities that these compounds can provide. Additionally, in animal nutrition, plant extracts are employed for several purposes, such as the enhancement of nutrient absorption, immune-boosting, antioxidant effects, resilience to diseases, etc. [21,22]. Thyme essential oil (TEO), grape seed extract (GSE), garlic oil (GAR), and capsicum oleoresin (CAP) are well known feed additives that, thanks to their compounds, allow for an enhancement of an animals’ health status and performance [23]. The two monoterpenes thymol and carvacrol are the most present in TEO and confer upon it a broad-spectrum antibacterial activity, in addition to antiviral, antifungal, antioxidant, and anti-inflammatory properties [24]. GSE is mainly employed for its antioxidant and anti-inflammatory activity provided by the polyphenols and flavonoids [25]. GAR contains organosulfur compounds, such as allicin, terpenoids, flavonoids, saponins, etc., and it is widely used in animal nutrition as a natural antimicrobial, antioxidant, and inflammation suppressor [26]. The main components of CAP are capsaicinoids, responsible for the pungent flavor of the extract and for its antimicrobial and antioxidant properties [27].

The aim of the study was to compare the immunometabolic modulation of two antibiotics and a panel of potential antibiotic alternatives during a *Salmonella* Enteritidis infection of a chicken macrophage-like cell line. In particular, we investigated the effect of an effective (gentamicin; GEN) and a non-effective (oxytetracycline; OXY) antibiotic versus four plant extracts (TEO, GSE, GAR, CAP).

## 2. Results

### 2.1. Minimal Inhibitory Concentrations of Antibiotics

The minimal inhibitory concentration (MIC) values of OXY and GEN are reported in Table 1. OXY was not able to inhibit the growth of *S.* Enteritidis up to the highest concentration tested (32 μg/mL), whereas GEN had a MIC correspondent to 4 μg/mL.

### 2.2. Kinome peptide array Analysis

The data elaborated from the kinome array exhibited changes in kinase activity of many peptides involved in the immunometabolic response of HD11 cells infected with *S.* Enteritidis and treated with antibiotics or plant extracts. Among all groups, gentamicin-treated cells showed the highest number of significant phosphorylation changes compared to the infected control, as shown in Table 2. 

#### 2.2.1. Kinome Peptide Array—Metabolic Modulation

The metabolic data related to glycolysis, TCA cycle, and oxidative phosphorylation from the kinome peptide array were reviewed.

**Figure 1:**
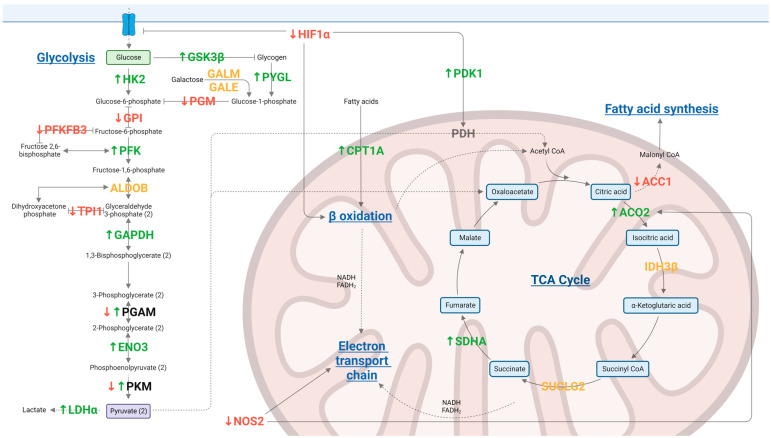
Summary of the main phosphorylation changes of significant metabolic peptides (*p* ≤ 0.05) of HD11 cells infected with *S.* Enteritidis and treated with gentamicin. ↑ protein, significantly activated; ↓ protein, significantly deactivated; protein, significantly phosphorylated but with no site information; ↓↑ protein, more than one site significantly phosphorylated with both activation and deactivation of the protein; inhibitor arrow indicates that phosphorylation of the protein leads to the inhibition of the linked pathway; normal arrow indicates that phosphorylation of the protein leads to the activation of the linked pathway; dashed arrow indicates that a product of a cellular metabolic pathway enters in a different metabolic pathway. Created with BioRender.com.

For gentamicin, phosphorylation changes of significant metabolic peptides are summarized in Figure 1. For all of the other groups, the main findings are described in Table 3.

#### 2.2.2. Kinome Peptide Array—Immune Modulation

**Figure 2:**
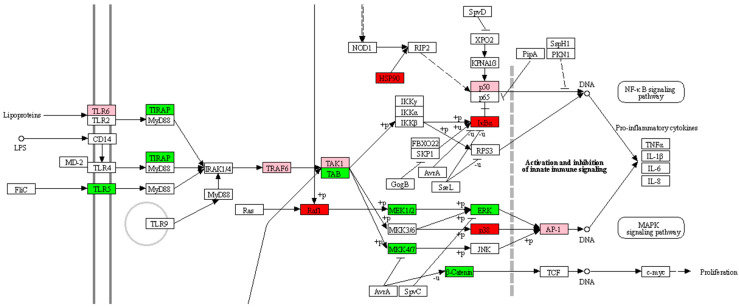
Section of *Salmonella* infection KEGG reference pathway (map05132) indicating the main phosphorylation changes of significant immune peptides (*p* ≤ 0.05) of HD11 cells infected with *S.* Enteritidis and treated with gentamicin. Green squares indicate significantly activated protein; red squares indicate significantly deactivated protein; pink squares indicate either no site information was available or more sites of the same protein were significantly affected with both activation/deactivation of the protein. Adapted with permission from [33]. 2023, Kanehisa Laboratories.

For the immune modulation, using the *Salmonella* infection KEGG reference pathway (map05132) [33], we focused on the TLR signaling cascade pathway, which leads to the promotion of the cellular immune response. For gentamicin, phosphorylation changes of significant immune peptides are described in Figure 2. For all the other groups, the main findings are described in Table 4.

### 2.3. Seahorse Metabolic Assay Analysis

**Figure 3:**
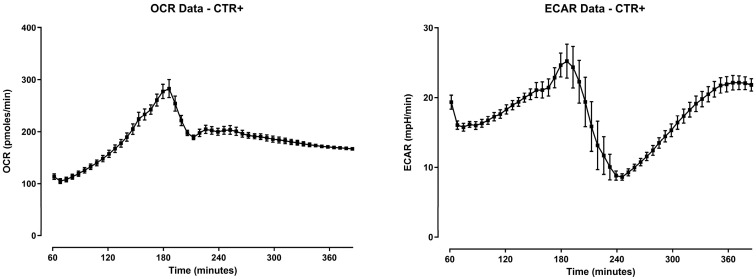
Oxygen consumption rate (OCR) and extracellular acidification rate (ECAR) of HD11 cells infected with *Salmonella* Enteritidis for 6 h. The reads start from 60 min since, during the first hour, the Seahorse XFp Analyzer requires an internal calibration. Data are expressed as the mean of all the experiments ± SEM (*n* = 18).

The mean trend of oxygen consumption rate (OCR) and extracellular acidification rate (ECAR) for the positive control, represented as the mean of all the experiments (*n* = 18), is reported in Figure 3. The infected HD11 cells without treatments showed an increase of both OCR and ECAR with a peak at 3 h post-infection. Then, a drop was observed for both the parameters, followed by the stabilization of OCR and a second peak of ECAR at the end of the 6 h.

**Figure 4:**
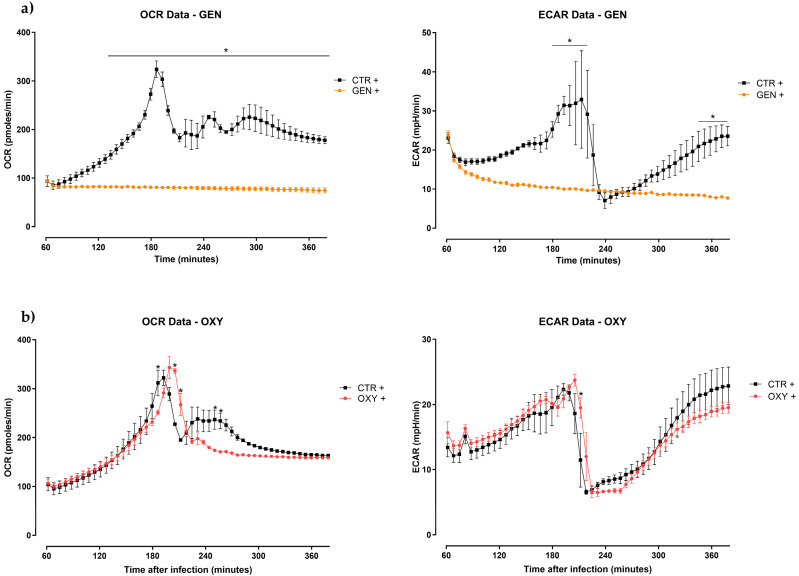
Oxygen consumption rate (OCR) and extracellular acidification rate (ECAR) of HD11 cells infected with *Salmonella* Enteritidis and treated with antibiotics ((**a**)—oxytetracycline (OXY) or (**b**)—gentamicin (GEN)) for 6 h. The reads start from 60 min since, during the first hour, the Seahorse XFp Analyzer requires an internal calibration. Data are expressed as the mean ± SEM (*n* = 3). Asterisks * indicate statistical significance with *p* ≤ 0.05.

**Figure 5:**
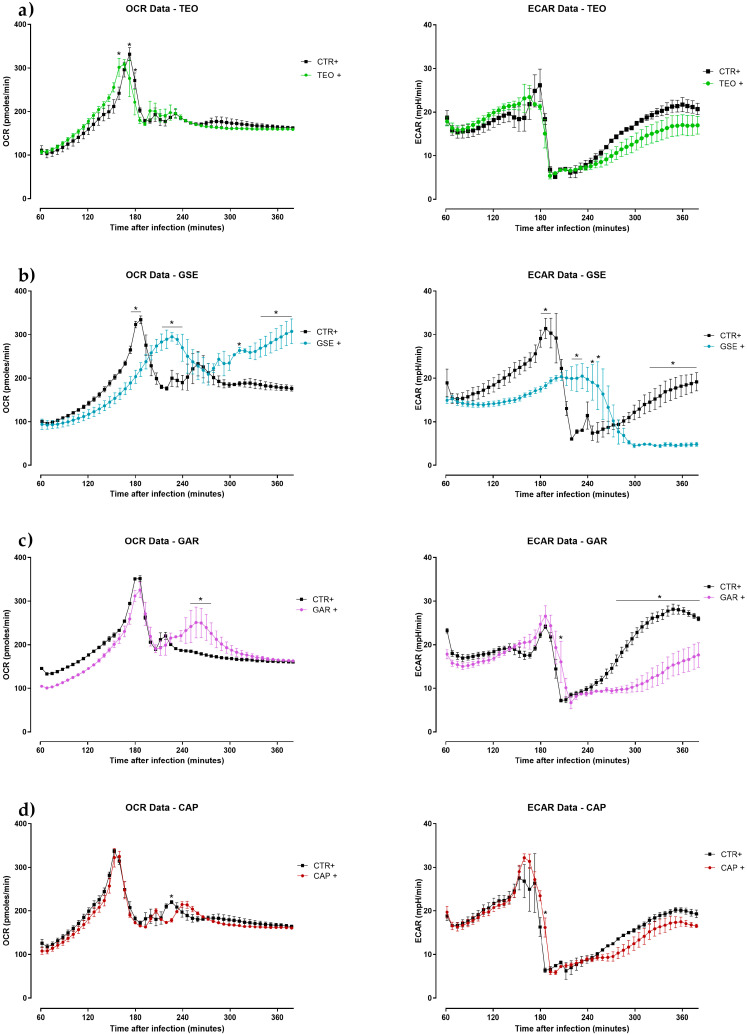
Oxygen consumption rate (OCR) and extracellular acidification rate (ECAR) of HD11 cells infected with *Salmonella* Enteritidis and treated with plant extracts ((**a**)—thyme essential oil (TEO), (**b**)—grape seed extract (GSE), (**c**)—garlic oil (GAR), or (**d**)—capsicum oleoresin (CAP)) for 6 h. The reads start from 60 min since, during the first hour, the Seahorse XFp Analyzer requires an internal calibration. Data are expressed as the mean ± SEM (*n* = 3). Asterisks * indicate statistical significance with *p* ≤ 0.05.

The graphs of OCR and ECAR of HD11 cells treated with antibiotics and plant extracts are reported in Figure 4a,b and Figure 5a–d, respectively.

After 2 h of infection, cells treated with GEN (Figure 4a) showed a significant difference in OCR, then, it was kept basal and constant during the entirety of the experiment. Additionally, ECAR was maintained basal during all the 6 h, showing significance at 3 h and at the end of the infection. The OXY + group (Figure 4b) exhibited a delayed peak of OCR after 3 h of infection, whereas a decrease of the same parameter compared to the CTR + was observed after 4 h. No differences in ECAR were reported. 

Little or no differences in OCR and ECAR were observed in cells treated with TEO (Figure 5a). The GSE + group showed a significant trend of increase in OCR and decrease in ECAR (Figure 5b). OCR increased between the 4 and 5 h of infection and ECAR decreased at the end of the experiment in the GAR + group (Figure 5c). No remarkable differences were reported for both OCR and ECAR with the CAP + group (Figure 5d).

### 2.4. Gene Expression Analysis

**Figure 6:**
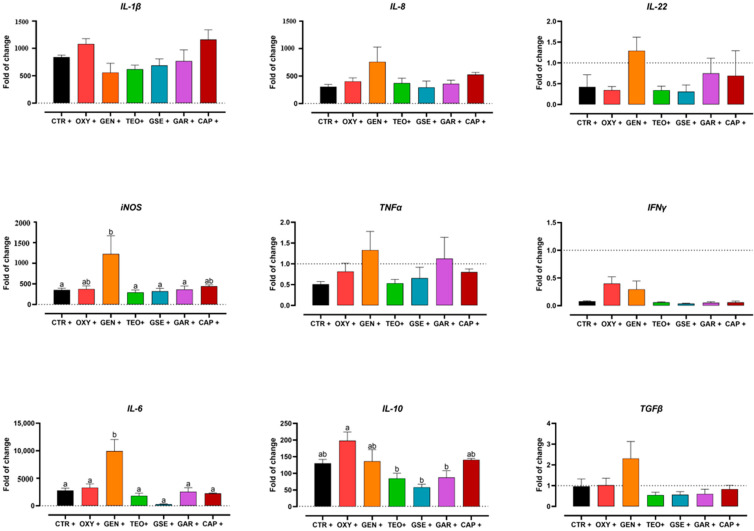
mRNA expression of *IL-1β*, *IL-8*, *IL-22*, *iNOS*, *TNFα*, *IFNγ*, *IL-6*, *IL-10*, and *TGFβ* of HD11 cells infected with *S.* Enteritidis and treated with antibiotics (oxytetracycline (OXY), gentamicin (GEN)) or plant extracts (thyme essential oil (TEO), grape seed extract (GSE), garlic oil (GAR), capsicum oleoresin (CAP)). Dashed lines represent the CTR—threshold, set as 1. Data are presented as mean ± SEM. Different letters indicate statistical significance with *p* < 0.05.

Gene expression results are shown in Figure 6. Among pro-inflammatory mediators, statistical significance among treatments was detected only for *iNOS*. HD11 cells treated with GEN significantly increased the expression of *iNOS* over the CTR +, TEO +, GSE +, and GAR + groups. *IL-1β* and *IL-8* were greatly upregulated compared to the non-infected control threshold, but with no statistical significance. The expression of *IL-6*, a dual-inflammatory cytokine, was significantly upregulated in GEN-treated cells compared to all other groups. Finally, among the two regulatory cytokines tested in this study (*IL-10* and *TGFα*), only *IL-10* was significantly upregulated in the OXY + group compared to the TEO +, GSE +, and GAR + groups.

## 3. Discussion

The role of pharmaceutical and natural substances in controlling foodborne diseases has been broadly surveyed through time. Other than the direct antimicrobial inhibitory or bactericidal action on the pathogen, the effect that these substances induce on the host is of growing interest. Certain classes of antibiotics, in fact, influence the host’s immune system, modulating its inflammatory response [15]. Tetracyclines exploit anti-inflammatory properties by decreasing several pro-inflammatory mediators of caspase pathways and matrix metalloproteinases [16]. Additionally, their empowerment of the innate immune system followed by an improved resolution of inflammation was reported, thanks to the enhancement of microbial-derived signaling and intestinal mucosal protection [17,18]. In this study, oxytetracycline did not show any effect in the Seahorse assay and any activity on matrix metalloproteinases or TLR signaling, as described by the kinome data; moreover, caspase 3 was activated (Supplementary Table S1), suggesting the contradiction of reported anti-inflammatory effect of tetracyclines. The aminoglycosides class has shown immunomodulatory properties, being able to suppress animals’ immune systems and inhibit neutrophil NAPDH oxidase [19,20]. As previously described, in this study, gentamicin-treated macrophages’ cells showed a deactivation of neutrophil NADPH oxidase factor 1 (data not shown). On the other hand, plant extracts are increasingly employed as feed additives thanks to their aromatic compounds, which confer them multiple properties. Thyme essential oil, grape seed extract, garlic oil, and capsicum oleoresin are renowned as antimicrobials, antioxidants, as well as anti-inflammatory compounds, even though their detailed mechanisms of modulation still need to be clarified [24,25,26,27,36]. In this study, the immunometabolic effect of oxytetracycline, gentamicin, and four plant extracts (thyme essential oil, grape seed extract, garlic oil, and capsicum oleoresin) were investigated on a chicken macrophages cell line during a *Salmonella* Enteritidis infection. Together with neutrophils, macrophages represent a first line of defense of the organism, being that their immune and metabolic profiles are strictly interconnected [37]. To accomplish this, the kinome peptide array was employed in order to deeply investigate the altered immunometabolic pathways. Then, the resulting data were corroborated through the Seahorse metabolic assay and the gene expression analysis.

To pursue the aim of the study, the sub-inhibitory dosages of the tested substances were used in order to exclude a direct antimicrobial effect. The selected concentrations, which are specified in Table 4, were therefore chosen based on the MIC assay for antibiotics, and from previous studies for all the plant extracts. The MIC assay of the antibiotics showed that according to Boothe et al. [38] and the National Antimicrobial Resistance Monitoring System for Enteric Bacteria (NARMS) [39], respectively, the *Salmonella* strain tested in this study was resistant to oxytetracycline (MIC not found up to 32 μg/mL) and susceptible to gentamicin (MIC = 4 μg/mL). 

The kinome data analysis revealed that the treatments showed variable changes in their kinase activity when compared to the infected control (Table 2). In particular, among all treatments, gentamicin had the highest number of altered proteins compared to the positive control. Gentamicin belongs to the aminoglycosides class; it is a bactericidal drug that targets and binds the 30S ribosome, inhibiting bacterial protein synthesis. Unlike all other treatments, gentamicin-treated cells showed significant changes in phosphorylation status of major proteins involved in glycolysis and the TCA cycle (Figure 1). Based on these findings, we conclude that the changes in the phosphorylation of these proteins resulted in the decreased ECAR and OCR described in the Seahorse assay (Figure 4a). Furthermore, activation of GSK indicates that glycogen synthase is inhibited, but the utilization of glycogen storage and its conversion in glucose happens thanks to PYG (Figure 1). Unlike other groups, treatment with gentamicin during *Salmonella* infection does not lead to inflammatory metabolic responses (increased glycolysis or pentose phosphate shunt), increased ROS as indicated by increased catalase activity (data not shown) and decreased HIF-1α and NOS, or heightened energy requirement supported by both kinome and seahorse metabolic flux data (Figure 1 and Figure 4a). However, in the gene expression analysis, *iNOS* expression was highly upregulated in the gentamicin group compared to most of the other treatments (Figure 6); this could highlight a discrepancy between kinome data that showed a deactivation of NOS2 (Figure 1). However, analogously to the *iNOS* trend, gentamicin also showed a great upregulation of *IL-6* expression, which could suggest its role as a pro-inflammatory cytokine (Figure 6). *IL-6* is a dual-inflammatory cytokine and its role in inflammation response is still not fully understood. It can exert an anti-inflammatory effect when expressed at low amounts and a pro-inflammatory effect in acute immune response [40]. Moreover, the release of pro-inflammatory mediators can be demonstrated by the cascade pathways that start with the TLRs activation during *Salmonella* infection (Figure 2). In fact, kinome analysis showed that kinase activity was increased for TLR1/3/5/6/8 in gentamicin-treated cells. TLRs activate the complex TIRAP-MyD88, leading to the activation of NF-kB and MAPK that, in turn, drive the release of pro-inflammatory mediators, like *iNOS* and *IL-6* [41,42,43,44]. In the gentamicin group, TIRAP kinase activity was increased by 25 times compared to the positive control and TIRAP-MyD88 cascade signaling activated IRAK, TRAF6 and TAK1. Concerning NF-kB signaling, we saw a decrease in kinase activity of IkBa (or NF-kB inhibitor alpha); this leads to the dissociation of the two subunits and their translocation in the nucleus for the initiation of inflammation [42,43]. For MAPK signaling, the activation of MEK1/2 and ERK, other than MKK4/7, was shown. As for NF-kB, the MAPK translocation to the nucleus initiates the inflammatory response [42]. 

Oxytetracycline is a protein synthesis inhibitor that belongs to the tetracyclines class. As gentamicin, it binds to the 30S ribosomal subunit but, unlike the other drug, its binding is reversible, possibly suggesting a weaker antibacterial effect. In fact, the *Salmonella* Enteritidis strain used in this study was resistant to oxytetracycline. The resistance phenomenon of *Salmonella* to oxytetracycline is common and previously reported in multispecies-isolated strains [45]. Considering the Seahorse assay (Figure 4b), the non-effective antibiotic does not have any particular effect on the metabolic side. The same can be said for the kinome data, as we observed significant changes in a very few immunometabolic proteins, most of whom had site activity which remains unknown (Table 3 and Table 4). The expression of *IL-10* has been found to be significantly higher in oxytetracycline compared to all the plant extracts other than capsicum oleoresin (Figure 6). In order to study the changes in phosphorylation exclusive to oxytetracycline, a Venn diagram was designed comparing the non-effective antibiotic and the three plant extracts (thyme essential oil, grape seed extract, and garlic oil). Then, the elements included exclusively in oxytetracycline were analyzed (Supplementary Figure S1). The increased expression of *IL-10* could be validated by the activation of CKIIα, which is involved in the binding of ATF1 and the activation of the ATF/CREB family of transcription factors [46]. The phosphorylation of CREB promotes the production of IL-10 in macrophages in response to TLR and C-type receptor signaling [47]. Additionally, the kinase activity of VEGFR-3, the receptor for vascular endothelial growth factors, was upregulated in oxytetracycline; it has been demonstrated that the signal driven by VEGFR-3 has been correlated with an increased expression of *IL-10* [48]. On the other hand, it has been reported that *IL-10* decreases caspase-3 activity [49], which, in our study, resulted in activation according to the kinome data. The peptide array results also suggested that the PI3K pathway is non-active because of the inhibition of PIK3R1 kinase activity. This would lead to the suppression of GSK3 inhibition by PI3K pathway and the consequent block of *IL-10* production by GSK3 [50]. No direct information about the kinase activity of GSK3 was found in the kinome data of oxytetracycline. Finally, the downregulation of IFNAR1 phosphorylation would lead to the increased expression of pro-inflammatory cytokines via JAK/STAT activation [51,52]. At the end, the increased expression of *IL-10* in the oxytetracycline group was not fully justified by the kinome data, but it must be considered that qPCR results do not provide information about the effective translation and production of the protein. Lastly, as mentioned above, the apoptotic effector caspase 3 was activated in oxytetracycline; this is reported to affect *Salmonella* infection by increasing the bacterial invasiveness of macrophages and dissemination [53], in addition to promoting apoptosis [54]. The same site that was activated in oxytetracycline (S150) was found to be significantly non-active in gentamicin-treated cells (Supplementary Table S1). This could explain the observed difference between the two treatments, also suggesting an anti-apoptotic role of gentamicin.

In this study, thyme essential oil did not influence the metabolic status of the cell during the bacterial challenge (Figure 5a). Similar to the Seahorse results, there were little metabolic differences observed in the kinome data (Table 3). There were changes in the phosphorylation of glycolytic and TCA proteins, including PFK, TPI, and Succinyl-CoA ligase. The little difference observed in OCR could be as a result of the changes in succinate metabolism. Further investigations are needed, since succinate could promote *Salmonella* survival into macrophages [55]. However, these differences did not induce any further effects, as identified by the Seahorse assay. Furthermore, the kinome-significant immune proteins and the cytokine gene expression did not show any noteworthy influence as well (Table 4 and Figure 6).

The number of changes in phosphorylation compared to the infected control highlights that grape seed extract had the highest count among the tested extracts and oxytetracycline (Table 2). As shown in Figure 5b, the cells were in an aerobic and less inflammatory status thanks to the grape seed extract, being still able to fight the challenge. The Seahorse assay showed a switch in metabolism around the 3 h mark, which resulted in a decrease in ECAR and an increase in OCR. The kinome peptide array data supported the changes observed (Table 3). The increase in OCR is facilitated by pyruvate kinase activity (to yield an ATP molecule) to promote pyruvate oxidation for TCA cycling. Increased OCR is also supported by decreased activity of HK2, which is known to decrease pyruvate and lactate flux without affecting the pyruvate to TCA fluxes [56]. Lastly, the increased phosphorylation of AMPK on its active site is known to increase catabolic energy generation via oxidative phosphorylation whilst switching off inefficient metabolism like glycogen metabolism [57]. Decreased glycogen metabolism is observed via decreased activity of GSK and PYG. To initiate the effective energy generation via oxidative phosphorylation, there needs to be an input of substrates into the TCA cycle; however, fatty acid metabolism appears to be inhibited/decreased (SIRT1, PPAR, ACC). Thus, pyruvate from glycolysis was used to fuel this initial switch. This is supported by the activation status of PFK, PGAM, and PKM. The activation of PKM can also be sustained through the phosphorylation of HSP90, as the kinome data show [34]. On the immunomodulation side, kinome exhibited the activation of numerous proteins involved in the activation of MAPK and NF-kB signaling (Table 4), even if the gene expression did not highlight any significant result, as shown in Figure 6.

The kinome data showed that cells treated with garlic oil exhibited increased changes in the phosphorylation of proteins involved in fatty acid oxidation (Table 3). Fatty acid oxidation can be used as an energy source to fuel the TCA cycle during low energy modes or decreased glucose/glycogen sources [58]. We observed an increase in OCR at the 4–5 h mark and a decrease in ECAR at the same time points (Figure 5c). However, the increased OCR was not sustained. We also observed deactivation of acetyl-CoA carboxylase, which leads to the increased accumulation of malonyl-CoA for fatty acid synthesis, thus reducing the input of fatty acid oxidation into the TCA cycle [59]. On the other hand, the decrease in ECAR/glycolysis may be explained by a decrease in glucose availability. We observed increased phosphorylation of PGYL and PHK, which may indicate increased activity to breakdown glycogen, which perhaps is an attempt to restore energy generation via glycolysis. The increased activities of PFK and PGAM are evidence of glycolysis in garlic oil-treated cells, which was not sustained. Immunometabolic changes induced by garlic oil are not potent enough for a favorable profile.

In this study, the immunometabolic status of the cell is not influenced by capsicum oleoresin during the bacterial challenge (Figure 5d and Figure 6). The kinomic profile, in fact, showed very little changes in protein involved in both immune and catabolic metabolism, including glycolysis (Table 3 and Table 4). Thus, the similarity between capsicum oleoresin and positive control seen in the metabolic flux assay and cytokine gene expression analysis is supported by the kinome data.

## 4. Conclusions

Based on these findings, we can conclude that:

- Gentamicin led to a hybrid phenotype of macrophages, supported by an activation of immune pathways with increase of pro-inflammatory mediators and, at the same time, a less inflammatory metabolic response. Thereby, the effective antibiotic could help the host respond to the bacterial insult, avoiding the overreaction of its metabolic processes.
- The non-effective antibiotic (oxytetracycline) had no influence on macrophages’ metabolic profile and its role in immunomodulation was not totally supported by the kinome data.
- Grape seed extract switched macrophages to a less inflammatory status by promoting TCA cycle and oxidative phosphorylation. Even if several immune pathways were active according to kinome data, no evidence of expression of pro-inflammatory proteins was found.
- Garlic oil affected glycolysis and the metabolic and inflammatory status of macrophages, which was not supported by the kinome analysis.
- Thyme essential oil and capsicum oleoresin did not affect the immunometabolic profile of chicken macrophages. A possible explanation may be that the concentration was too low to elicit a response.

In contrast to the metabolic data obtained in the study, the results of the gene expression analysis suggested a greater homogeneity of the treatments, since a few statistical differences were found. This could be explained by the common panel of cytokines investigated with gene expression that should perhaps be implemented with more specific targets, especially for the plant extracts, for which the complete mechanisms of action still need to be clarified. Moreover, further investigations about the specific macrophages’ phenotype are needed in order to confirm the results obtained with this study.

## 5. Materials and Methods

### 5.1. Cell Line, Bacterial Strain, and Culture Conditions

HD11 cells were obtained from the laboratory of Dr. Mark Parcells (University of Delaware). The cells were maintained at 37 °C, 5% CO_2_, and 95% humidity in Iscove’s Modified Dulbecco’s Media (IMDM; GE Life Sciences, Logan, UT, USA) with 10% fetal bovine serum (Midsci, Valley Park, MO, USA) and 1% 1.5 mM L-glutamine containing penicillin and streptomycin (Gibco, Grand Island, NY, USA).

*Salmonella* Enteritidis, resistant to nalidixic acid and novobiocin, was donated by Dr. Haiqi He, US Department of Agriculture Research Service. The strain was conserved at −80 °C in tryptic soy broth (TSB; Becton, Dickinson and Company, Sparks, MD, USA) and supplemented with 20% (*v*/*v*) glycerol. The day prior to the experiments, it was thawed and cultured overnight in TSB supplemented with 25 μg/mL novobiocin and 20 μg/mL nalidixic acid (Sigma-Aldrich, St. Louis, MO, USA) at 37 °C.

### 5.2. Chemical and Test Solutions

OXY and GEN were purchased from Alpha Aesar (Alpha Aesar, Ward Hill, MA, USA) and Sigma-Aldrich (Sigma-Aldrich, St. Louis, MO, USA), respectively. TEO and CAP were obtained from Frey&Lau (Frey + Lau GmbH, Henstedt-Ulzburg, Germany), GSE was obtained from Layn Natural Ingredients (Guilin Layn Natural Ingredients Corp., Shanghai, China), and GAR from Lluch Essence (Lluch Essence S.L.U, Barcelona, Spain).

Stock solutions of antibiotics (256 μg/mL) and plant extracts (40,000 μg/mL) were prepared in water and ethanol, respectively. The solutions were filter-sterilized and conserved at +4 °C or −20 °C (GEN).

All the substances in this study were tested at non-inhibitory concentrations. The experimental dosages of plant extracts were chosen based on previous internal studies, whereas dosages of OXY and GEN were determined by performing a MIC assay using a broth microdilution method in 96-well microtiter plates. The antibiotics were tested at final concentrations ranging from 32 to 0.25 μg/mL (2-fold dilutions) in TSB at pH 6.5. The inoculum was prepared from an overnight culture of *Salmonella* Enteritidis diluted up to 10^5^ CFU/mL. After 24 h of incubation at 37 °C, the MIC of the antibiotics was defined as the lowest concentration that resulted in null absorbance (600 nm) registered with a spectrophotometer (Molecular Devices Microplate Reader SpectraMax Plus; Molecular Devices, San Jose, CA, USA). For the following experiments, non-inhibitory concentrations of the antibiotics were selected.

### 5.3. Salmonella Infection of HD11 Cells

The day of the infection, the overnight culture of *Salmonella* Enteritidis was passaged in fresh TSB and incubated for 4 h at 37 °C. HD11 cells were seeded on the appropriate culture plates (see subsections below) and allowed to adhere to the wells for at least 2 h. Then, the concentration of *Salmonella* Enteritidis during the exponential phase was calculated using optical density, as reported by Perry et al. [14], and the bacterial suspension was diluted in order to infect cells with a multiplicity of infection (MOI) of 10. At the same time, cells were treated in triplicate, as reported in Table 5, diluting the compounds to their final concentration in either IMDM with 10% fetal bovine serum, for gene expression and kinome analysis, or Seahorse media (XF DMEM medium with 5 mM HEPES, pH 7.4; Agilent Technologies, Santa Clara, CA, USA) containing 1% 200 mM glutamine and 1% 100 mM sodium pyruvate. The total time of infection for all the experiments was 6 h.

#### 5.3.1. Kinome Peptide Array Analysis

For the kinome experiment, 2.5 × 10^6^ cells/well were seeded in 6-well microtiter plates.

At the end of the 6 h of infection, the supernatants were discarded, the cells were gently washed with PBS and then trypsinized for 5 min at 37 °C. After trypsinization, the enzyme was inhibited with IMDM with 10% fetal bovine serum and the detached cells were centrifuged at 800× *g* for 3 min. The supernatants were discarded, the pellets were flash frozen in liquid nitrogen, and then stored at −80 °C.

The protocol followed for this experiment is reported by Arsenault et al. [60]. In brief, the pelleted cells were thawed and incubated with a lysis buffer after being thoroughly vortexed. The Pierce™ Rapid Gold BCA Protein Assay Kit (Thermo Fisher Scientific, Waltham, MA, USA) was performed using 10 uL of the lysate in order to eventually dilute the samples to the same concentration. Then, the lysates were centrifuged at the maximum speed for 10 min. 10 uL of an activation mix containing 500 μM of ATP were added to 90 uL of supernatants: the suspension was well vortexed and an aliquot of 75 uL was applied to a glass peptide array (JPT Peptide Technologies, Berlin, Germany) incubated in a humid sealed container at 37 °C for 2 h. At the end of the incubation, the arrays were washed, stained in ProQ Diamond Phosphoprotein Stain (Life Technologies, Carlsbad, CA, USA) for 1 h, and then destained using a solution containing 20% acetonitrile and 50 mM sodium acetate. A Tecan PowerScanner™ (Tecan Systems, San Jose, CA, USA) was used to detect dye fluorescence at 532 to 560 nm with a 580-nm filter. The outcoming images were gridded using GenePix Pro software (version 7.2.29 1, Molecular Devices, CA, USA) and data were analyzed with Platform for Intelligent Integrated Kinome Analysis (PIIKA 2.5) [61], followed by other online databases, like STRING [62], PhosphoSitePlus^®^ v. 6.6.0.4 [63], KEGG pathways [33], and Venny v. 2.1 [64].

#### 5.3.2. Seahorse Metabolic Assay Analysis

For the seahorse experiment, 5 × 10^4^ cells/well were seeded in Seahorse mini culture plates (Agilent Technologies, Santa Clara, CA, USA), excluding wells A and H. The wells on the side of the plate were filled with 400 μL of double-deionized water.

The calibration plate of the Seahorse XFp Analyzer (Agilent Technologies, Santa Clara, CA, USA) was prepared as reported by Perry et al. [14] to calibrate the machine prior to the experiment. In brief, at the start of the infection, cells were incubated at 37 °C in a non-CO_2_ incubator for 1 h. During this time, the calibration of the instrument was carried out and, at the end of the incubation time, the calibration plate was replaced by the experimental plate. During the following 5 h, the ECAR and the OCR were measured every 6 min for a total of 50 measurements.

#### 5.3.3. Gene Expression Analysis

For the gene expression experiment, 10^6^ cells/well were seeded in 24-well microtiter plates.

At the end of the 6 h of infection, the supernatants were discarded and the wells were gently washed with PBS. The cells were then trypsinized for 5 min at 37 °C. After trypsinization, the enzyme was inhibited with IMDM with 10% fetal bovine serum and the detached cells were centrifuged at 800× *g* for 3 min. The supernatant was discarded and the pellet was resuspended in RNeasy^®^ Mini Kit (Qiagen; Hilden, Germany) lysis buffer supplemented with 1% β-mercaptoethanol (Fair Lawn, NJ, USA) and vortexed thoroughly for 30 s. Lysed cells were flash frozen and stored at −80 °C.

For RNA extraction, the lysate was thawed at room temperature and the animal cell protocol given by the RNeasy^®^ Mini Kit was followed. RNA yield and quality were verified spectrophotometrically using A230, A260, and A280 nm measurements (NanoDrop™, Thermo Fisher Scientific, Waltham, MA, USA). The extracted RNA was converted into cDNA using GeneAmp™ Fast PCR Master Mix (Applied Biosystems, Waltham, MA, USA) according to manufacturer’s instructions.

Gene expression analysis was performed with a real-time PCR using QuantStudio 3 Real-Time PCR Systems (Thermo Fisher Scientific, Waltham, MA, USA). The reaction contained 5 μL of 2X PowerUp™ SYBR™ Green Master Mix (Thermo Fisher Scientific, Waltham, MA, USA), 300 or 600 nM of each primer listed in Table 6, 2 μL of 5 ng/μL cDNA, and nuclease-free water, up to the final volume of 10 μL. The samples were analyzed under the following conditions: 2 min at 95 °C, followed by 40 cycles of 95 °C for 1 s and 60 °C for 30 s. The specificity of each reaction was evaluated by melting-curve analysis with 0.15 °C/s heating rate from 60 up to 95 °C.

mRNA expression was normalized using 28S as a housekeeping gene. After determining the threshold cycle (Ct) for each gene, the relative changes in mRNA expression were calculated using the 2^−ΔΔCt^ method [65], comparing all the groups with the CTR −.

**Table 6:** List of primers for real-time PCR.

Gene	Sequence (5′ → 3′)	Accession Number	Reference
28S	F: GGCGAAGCCAGAGGAAACT’ R: GACGACCGATTGCACGTC	X59733	[66]
*IL-1β*	F: GCTCTACATGTCGTGTGTGATGAG R: TGTCGATGTCCCGCATGA	AJ245728	[66]
*IL-6*	F: GCTCGCCGGCTTCGA R: GGTAGGTCTGAAAGGCGAACAG	AJ250838	[66]
*IL-8*	F: GCCCTCCTCCTGGTTTCAG R: TGGCACCGCCAGCTCATT	AJ009800	[66]
*IL-10*	F: CATGCTGCTGGGCCTGAA R: CGTCTCCTTGATCTGCTTGATG	AJ621614	[67]
*IL-22*	F: CAGGAATCGCACCTACACCT R: CGGTTGTTCTCCCTGATGTT	NM_001199614	[68]
*iNOS*	F: TTGGAAACCAAAGTGTGTAATATCTTG R: CCCTGGCCATGCGTACAT	NM_204961	[69]
*TFGβ*	F: AGGATCTGCAGTGGAAGTGGAT R: CCCCGGGTTGTGTGTTGGT	M31160	[66]
*TNFα*	F: CCCTACCCTGTCCCACAACC R: TGGGCGGTCATAGAACAGCA	XM_046927265.1	
*IFN* *γ*	F: GTGAAGAAGGTGAAAGATATATCATGGA R: GCTTTGCGCTGGATTCTCA	Y07922	[66]

The *TNFα* primers were designed by Dr. Yihang Li (University of Delaware).

### 5.4. Statistical Analysis

For the kinome peptide array, a one-sided paired t-test between infected treatments and positive control values was performed for each peptide via PIIKA 2.5 [61]. Both the Seahorse metabolic assay and the gene expression data were analyzed using Graphpad Prism v. 9.4.1 (GraphPad Software Inc., San Diego, CA, USA). For the first one, an ordinary two-way ANOVA was performed, followed by a Šidák’s multiple comparison test, to compare the infected treatments and positive control values. For the gene expression analysis, an ordinary one-way ANOVA was performed, followed by a Tukey’s multiple comparison test, to compare every group with each other. 

## Figures and Tables

**Table 1:** Minimal inhibitory concentration (MIC) of oxytetracycline and gentamicin.

Antibiotic	Range of Concentration Tested (μg/mL)	MIC (μg/mL)
Oxytetracycline	0.25–32	>32
Gentamicin	0.25–32	4

**Table 2:** Number of changes in phosphorylated proteins of treated groups in comparison to infected control (*p* ≤ 0.05).

Treatment Groups Compared to Infected Control	Number of Changes in Phosphorylation
Gentamicin	540
Grape seed extract	117
Thyme essential oil	69
Oxytetracycline	68
Garlic oil	66
Capsicum oleoresin	52

**Table 3:** Changes in the phosphorylation of significant metabolic peptides (*p* ≤ 0.05) of HD11 cells infected with *S.* Enteritidis and treated with oxytetracycline or plant extracts.

Group	Protein and Phosphorylation Change ^1^	Status of the Protein ^2^
Oxytetracycline	PRKAB2 ↑	Active
	PGAM1 ↑	Non active
	HK1 ↑	Active
	ACOT12 ↓	na
	PKLR ↓	Non active (liver) [28]
	CAT ↓	Non active
Thyme essential oil	TPI1 ↓	Non active [29]
	ACSL6 ↓	na
	ACSL5 ↓	na
	SUCLA2 ↓	na
	ACAA1 ↑	na
	PGAM1 ↑	Active
	PFKP ↑	Non active
	HK1 ↓	Active
	HK2 ↑	Active
	PFKL ↓	Non active
Grape seed extract	PFKFB1 ↓	Non active [30]
	PYGL ↓	Active
	HK2 ↓	Active
	PGAM1 ↑	Active
	PYGM ↓	Active
	PFKP ↓	Non active [31]
	PKM ↑	Active
	CAT ↓	Active or non active [32]
	PKLR ↓	Non active (liver) [28]
Garlic oil	PFKP ↓	Non active [31]
	PHKA2 ↑	Active
	PGAM1 ↑/↑	Active/na
	PYGL ↑	Active
Capsicum oleoresin	LDHA ↓	Active
	PKLR ↓	Non active
	PRKAA1 ↓	Non active
	CPT1A ↑	Active
	ALDOB ↓	na

^1^ ↑, significantly more phosphorylated and consequence of phosphorylation unknown; ↓, significantly less phosphorylated and consequence of phosphorylation unknown; ↑, significantly more phosphorylated on an active site; ↓, significantly less phosphorylated on an active site; ↑, significantly more phosphorylated on an inhibitory site. Where more than one arrow is indicated, more sites of the same protein were significantly affected. ^2^ The status of the protein was assumed based on the phosphorylation change and the function of the affected site (active/inhibitory) or, based on existing literature, where no site information was available.

**Table 4:** Changes in the phosphorylation of significant immune peptides (*p* ≤ 0.05) of HD11 cells infected with *S.* Enteritidis and treated with oxytetracycline or plant extracts.

Group	Protein and Phosphorylation Change ^1^	Status of the Protein ^2^
Oxytetracycline	MAPK14 ↑	
MAPK14 ↑	MAP2K4 ↑	Active
	MAPK14 ↑	Active
Grape seed extract	CHUK ↑	Active
	HSP90AB1 ↑	Active
	HSP90B1 ↑	Active [34]
	MAP2K1 ↓	Non active [35]
	MAP2K4 ↑	Active
	MAP3K7 ↑	Active
	MAPK14 ↑	Active
	RAF1 ↓	Active
Garlic oil	MAPK1 ↑	Active
	RAF1 ↑	Active
Capsicum oleoresin	MAP2K4 ↑	Non active
	MAPK1 ↑	Active

^1^ ↑, significantly more phosphorylated and consequence of phosphorylation unknown; ↓, significantly less phosphorylated and consequence of phosphorylation unknown; ↑, significantly more phosphorylated on an active site; ↑, significantly more phosphorylated on an inhibitory site; ↓, significantly less phosphorylated on an inhibitory site. Where more than one arrow is indicated, more sites of the same protein were significantly affected. ^2^ The status of the protein was assumed based on the phosphorylation change and the function of the affected site (active/inhibitory) or, based on existing literature, where no site information was available.

**Table 5:** Experimental designs describing treatment groups, the concentrations of the tested compound, and in which experiments they were carried out.

Group (*n* = 3)	Concentration of Tested Compound	Infection ^1^	Experiments ^2^
Negative control (CTR –)	None	–	G
Positive control (CTR +)	None	+	K/S/G
Oxytetracycline (OXY)	32 μg/mL	+	K/S/G
Gentamicin (GEN)	2 μg/mL	+	K/S/G
Thyme essential oil (TEO)	10 μg/mL	+	K/S/G
Grape seed extract (GSE)	100 μg/mL	+	K/S/G
Garlic oil (GAR)	10 μg/mL	+	K/S/G
Capsicum oleoresin (CAP)	10 μg/mL	+	K/S/G

^1^ +, infected with *S*. Enteritidis at MOI 10; –, not infected. ^2^ G, gene expression analysis; K, kinome assay; S, seahorse assay.

## Data Availability

Data available on request due to restrictions e.g., privacy or ethical.

## Supplementary Materials

The following supporting information can be downloaded at: https://www.mdpi.com/article/10.3390/antibiotics12020357/s1, Table S1: Changes in the phosphorylation of caspase 3 (CASP3; *p* ≤ 0.05) of HD11 cells infected with S. Enteritidis and treated with antibiotics or plant extracts; Figure S1: Venn diagram of oxytetracycline, thyme essential oil, grape seed extract, and garlic oil, and changes in the phosphorylation of significant selected immune peptides (*p* ≤ 0.05) exclusive of oxytetracycline.
