# Supplementary material for: A Comparison of the Immunometabolic Effect of Antibiotics and Plant Extracts in a Chicken Macrophage-like Cell Line during a Salmonella Enteritidis Challenge
Source: Antibiotics (Basel). 2023 Feb 8;12(2):357. doi: 10.3390/antibiotics12020357 (PMC9952652; doi:10.3390/antibiotics12020357)

Supplementary Table S1: Changes in the phosphorylation of caspase 3 (CASP3; $p \leq 0.05$) of HD11 cells infected with *S.* Enteritidis and treated with antibiotics or plant extracts.

Group	Protein and phosphorylation change[1]	Status of the protein[2]
Oxytetracycline	CASP3 ↓	Active
Gentamicin	CASP3 ↑	Non active
Thyme essential oil	No significant changes observed	-
Grape seed extract	No significant changes observed	-
Garlic oil	No significant changes observed	-
Capsicum oleoresin	No significant changes observed	-

[1] ↑, significantly more phosphorylated on an inhibitory site; ↓, significantly less phosphorylated on an inhibitory site.

[2] The status of the protein was assumed based on the phosphorylation change and the function of the affected site (active/inhibitory).

Supplementary Figure S1: Venn diagram of oxytetracycline, thyme essential oil, grape seed extract, and garlic oil, and changes in the phosphorylation of significant selected immune peptides (p ≤ 0.05) exclusive of oxytetracycline.

Protein and phosphorylation change[1]	Status of the protein[2]
CARD11 ↑	Active
CASP3 ↓	Active
CKIIα ↑	Active
VEGFR-3 ↑	Active
IFNAR1↓	Active
PIK3R1 ↓	Non active

[1] ↑, significantly more phosphorylated and consequence of phosphorylation unknown; ↓, significantly less phosphorylated and consequence of phosphorylation unknown; ↑, significantly more phosphorylated on an active site; ↓, significantly less phosphorylated on an active site; ↑, significantly more phosphorylated on an inhibitory site; ↓, significantly less phosphorylated on an inhibitory site.

[2] The status of the protein was assumed based on the phosphorylation change and the function of the affected site (active/inhibitory) or, based on existing literature where no site information was available..

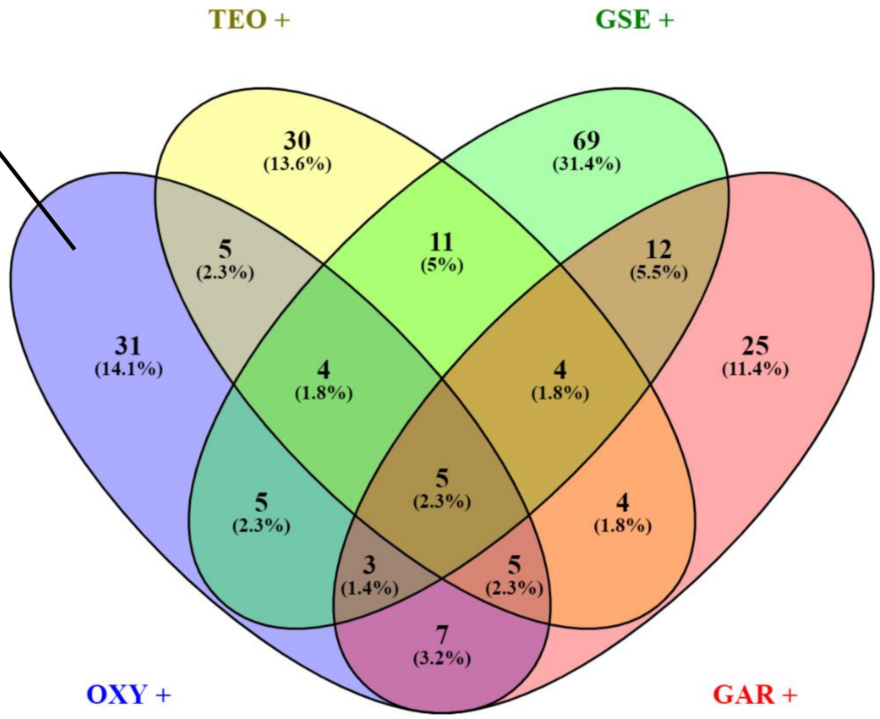

Supplement: Supplementary file 1 [file antibiotics-12-00357-s001.zip › antibiotics-2149617-supplementary.pdf]
